# Association Between Patient Demographics and Smoldering Multiple Myeloma Progression to Multiple Myeloma: A SEER-Medicare Data Analysis

**DOI:** 10.1016/j.clml.2025.06.016

**Published:** 2025-06-22

**Authors:** Poy Theprungsirikul, Rong Wang, Ishfaq Ahmad, Natalia Neparidze, Xiaomei Ma, Su-Hsin Chang, Shi-Yi Wang

**Affiliations:** 1 Department of Internal Medicine, Section of Hematology, Yale School of Medicine, New Haven, CT; 2 Cancer Outcomes, Public Policy, and Effectiveness Research (COPPER) Center, Yale University, New Haven, CT; 3 Department of Chronic Disease Epidemiology, Yale School of Public Health, New Haven, CT; 4 Knight Cancer Institute, Oregon Health & Science University, Portland, OR; 5 Division of Public Health Sciences, Department of Surgery, Washington University School of Medicine in St. Louis, St. Louis, MO

**Keywords:** Multiple myeloma, Patient characteristics, Progression, Smoldering multiple myeloma

## Abstract

The association between demographic factors and progression from smoldering multiple myeloma (SMM) to symptomatic multiple myeloma is unclear. In this analysis of Surveillance, Epidemiology, and End Results-Medicare data, we sought to determine the association between age/gender/race and SMM progression. We identified 1235 SMM patients. After adjustment, only age, but not race and gender, is negatively associated with SMM progression.

**Background:**

While several risk stratification models for smoldering multiple myeloma (SMM) to symptomatic multiple myeloma (MM) progression have been developed, the association between patient demographics, such as race, gender, and age, and SMM progression is not well understood.

**Methods:**

Analyzing surveillance, epidemiology, and end results (SEER)-Medicare data, we applied a previously developed algorithm to identify patients with SMM diagnosed between 2007 and 2019. We used noncancer patients from the 5% random sample of Medicare beneficiaries as the controls. Cox proportional hazards models were applied to assess the association between race/gender/age and the development of hypercalcemia, renal failure, anemia and bone disease among SMM patients and the controls. We applied bootstrapping to calculate the estimates hazard ratios (aHRs) and 95% confidence intervals (CIs) of progression among SMM patients, adjusting for that of the noncancer controls.

**Results:**

Out of 1235 identified SMM patients (median age 75 years, White 76.7%), 856 (69.3%) of them progressed to symptomatic MM. Race (Black vs. White aHR = 0.82, 95% CI: 0.65-1.01) and gender (male vs. female aHR = 0.99, 95% CI: 0.86-1.13) were not significantly associated with SMM progression. Only age was negatively associated with SMM progression (75-79 years vs. 66-69 years aHR = 0.71, 95% CI: 0.58-0.87; 80-84 years vs. 66-69 years aHR = 0.59; 95% CI: 0.46-0.74; and ≥ 85 years vs. 66-69 years aHR = 0.59; 95% CI: 0.45-0.75).

**Conclusion:**

This analysis provided insight into important parameters for MM natural history modeling by demonstrating that only age, but not race and gender, is negatively associated with SMM progression.

## Introduction

Smoldering multiple myeloma (SMM) is an asymptomatic clonal plasma cell disorder with a risk of progression to active multiple myeloma (MM), which accounts for approximately 10% of hematologic malignancies.^1 , 2^ It is defined by the presence of a serum monoclonal protein of ≥ 3 g/dL (or ≥ 500 mg/24 h in urine or both) and/or 10% to 60% clonal bone marrow plasma cells with no evidence of end-organ damage (i.e., hypercalcemia, renal failure, anemia, or lytic bone lesions) or other myeloma-defining events.^3 , 4^ SMM is a rare disorder. For instance, the data from the Swedish Myeloma Registry revealed an age-standardized incidence of 0.44 cases per 100,000 individuals based on incidentally diagnosed SMM.^5^ In addition, the nationwide iStopMM screening study in Iceland demonstrated an SMM prevalence of 0.53% in individuals aged 40 years or older, with a higher prevalence in men (0.67%) than in women (0.39%).^6^ However, the information on incidence and prevalence of SMM at the population level in the United States is still limited. Analyzing data from the National Cancer Data Base (NCDB), Ravindran et al.^7^ estimated the incidence of SMM in the United States to be 0.9 cases per 100,000 individuals, though NCDB does not represent a true population-level dataset.

SMM is a heterogeneous clinical entity, and in comparison to monoclonal gammopathy of undetermined significance (MGUS), SMM carries a significantly higher risk of progression to MM with a 10% annual risk of progression to MM within the first 5 years after diagnosis, a 3% annual risk in the following 5 years, and a 1% annual risk after ten years.^2^ Outcomes in patients diagnosed with SMM can vary significantly; some individuals may never progress or experience very slow progression to symptomatic MM, while others may progress quickly.^8 , 9^ It is estimated that 25% of SMM patients never progress to symptomatic disease.^9^ Accurately classifying patients as having either indolent or aggressive SMM is crucial as those at high risk of progressing to symptomatic MM may benefit from early intervention, while low-risk patients can avoid unnecessary treatment.

Several risk stratification models have been proposed to predict the likelihood of SMM progressing to symptomatic MM.^10–14^ However, there is some discordance among these risk models.^15 , 16^ Furthermore, these models primarily focused on clinical and molecular cytogenetic parameters.^10–14^ Although some studies explored the links between demographic factors and the risk of progression from precursor states to MM, many of these studies grouped SMM with MM or MGUS with SMM.^14 , 17–21^ Li et al.^22^ showed in their meta-analysis that advanced age, female gender, and high body mass index (BMI) may be associated with the progression from MGUS to MM, while race was not statistically significantly linked to the risk. Yet, little is known regarding the relationship between demographic factors and the risk of progression from SMM to symptomatic MM.

In this study, we sought to examine the relationship between demographics and SMM progression to symptomatic MM. Specifically, we aimed to assess whether the progression from SMM to symptomatic MM varies based on race, gender, and/or age using the population-based Surveillance, Epidemiology and End Results (SEER)-Medicare data. Gaining a better understanding of how the progression from SMM to symptomatic MM differs by race, gender, and/or age could provide valuable insights into the impact of each factor on progression and aid in the development of MM natural history modeling to inform MM prevention strategies.

## Methods

### Study Design, Data and Statistical Analysis

We conducted SEER-Medicare data analysis to estimate hazard ratios (HRs) of SMM progression to symptomatic MM by race, gender, and/or age groups. SEER-Medicare links cancer records of Medicare beneficiaries from SEER registries with Medicare enrollment and claims data. The SEER registries cover approximately 48% of the United States population.^23^ The SEER-Medicare database links patient-level information on incident cancer diagnoses reported to the SEER registries with a master file of Medicare enrollment and claims for inpatient, outpatient, physician services, hospice care, home health agencies, durable medical equipment, and prescription drugs.^23^ The Yale Human Investigation Committee determined that the study did not directly involve human subjects.

We identified patients with first primary MM (International Classification of Diseases [ICD] for Oncology-3rd edition, 9732) who (1) were diagnosed between 2007 and 2019, (2) ages ≥ 66 years at diagnosis, and (3) had continuous Parts A and B enrollment and were not a Health Maintenance Organization (HMO) member from 12 months before diagnosis to end of follow-up (death or December 31, 2020, whichever came first). Additionally, we excluded patients who were identified by death certificates or autopsy only. Symptomatic MM is defined by a published algorithm.^24^ In brief, we identified CRAB symptoms (hypercalcemia, renal failure, chronic kidney disease, anemia, pathologic fracture and fracture of vertebral column) from inpatient, outpatient and carrier claims within 6 months before and after MM diagnosis. As these conditions are not specific for MM, we further excluded non-MM related conditions based on proximity to diagnosis and the presence of other possible causes. Specifically, we excluded: (1) hypercalcemia if hyperparathyroidism co-existed, (2) vertebral fractures if osteoporosis co-existed, and (3) anemia or chronic kidney disease if the patient also had anemia or chronic kidney disease more than 6 months before MM diagnosis.^24^ SMM is defined by absence of CRAB symptoms and no claims of MM treatment within 6 months of MM diagnosis. MM treatment was determined by using the Medicare claims for inpatient, outpatient, provider, and prescription coverage using the relevant International Classification of Diseases (ICD) and Healthcare Common Procedure Coding System (HCPCS) codes for injectable drugs and generic names for prescription drugs. MM treatment was defined as receipt of any of the following within 6 months after MM diagnosis: belantamab, bendamustine, bortezomib, carfilzomib, carmustine, cisplatin, cyclophosphamide, daratumumab, doxorubicin, elotuzumab, etoposide, isatuximab, ixazomib citrate, lenalidomide, melphalan, panobinostat, pomalidomide, selinexor, thalidomide, vincristine, unspecified antineoplastic chemotherapy or immunotherapy, or autologous or allogeneic stem-cell transplant.^25^ To define SMM progression, we used claims to identify treatment from 7 months after diagnosis to end of follow-up or MM mortality.

The primary outcome of interest was the progression to symptomatic MM. We ended follow-up at death, end of study (December 31, 2020), or 5 years after the beginning of the follow-up, whichever came first. We obtained data on the following patient characteristics: race, gender, age at diagnosis, marital status, residential region, state buy-in (as a proxy marker for socioeconomic status), and percentage of population below poverty at the census tract level. We constructed the Elixhauser comorbidity score and frailty index by searching for ICD-9 and ICD-10 diagnosis codes in the 12 months prior to SMM diagnosis that appeared on any inpatient claims or at least 2 outpatient/physician claims with an interval exceeding 30 days.^26–29^ We used Cox proportional hazard regression models to describe the associations between race, gender, and/or age and progression to symptomatic MM.

Acknowledging that there may be inherent differences regarding the associations between race, gender and/or age and the development of CRAB symptoms, we used noncancer patients from the 5% random sample of Medicare beneficiaries residing in the same areas as the control group. Each beneficiary identified as an SMM “case” was 1:1 frequency-matched to a beneficiary without cancer who served as a “control.” We created an index date for each “control” using the first day of a randomly selected month and year between 2007 and 2019 in which the control was alive and met the same enrollment criteria used for cases (patients with SMM) except for SMM diagnosis. “Cases” and “controls” were matched by age at the time of diagnosis for cases and the index date for controls, race (white/black), gender, comorbidity (yes/no), and year of diagnosis/index date. Cox proportional hazard regression models were applied to assess factors on the development of CRAB symptoms and to estimate the adjusted hazard ratios (aHRs) and 95% confidence intervals (CIs) for the control group. Both multivariable models included race, demographics, comorbidities, and socioeconomic factors, as well as the year of SMM/pseudo-SMM diagnosis. Upon getting the aHRs of MM/pseudo-MM progression by race, gender and/or age, we then used bootstrapping to calculate the estimates related to these factors, adjusting for the noncancer control aHRs. Statistical analysis was performed using SAS Version 9.4 (SAS Institute Inc., Cary, NC) and R, version 4.3.1 (R Project for Statistical Computing).

## Results

A total of 1235 patients were included in the analysis (Table 1). Median age at SMM diagnosis was 75 years. In the cohort, the majority of patients were female (50.4%), White (76.7%), in age group 70 to 74 years at SMM diagnosis (27.6%), had Elixhauser score 1 to 2 (41.2%), not frail (73%), from the Northeast region (42.4%) and had no state buy-in (84%).

A total of 856 out of 1,235 SMM patients (69.3%) progressed to symptomatic MM.

**Figure 1:**
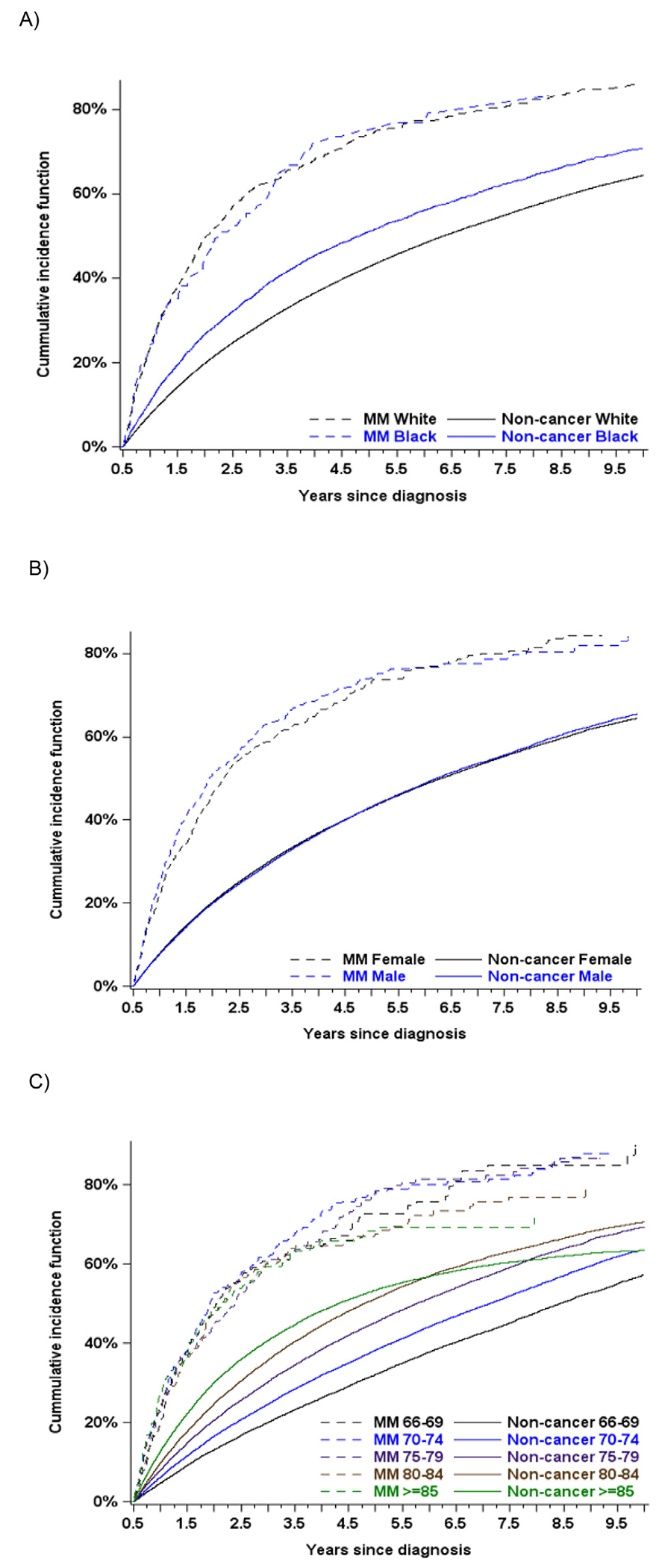
Cumulative incidence of progression from smoldering multiple myeloma to symptomatic multiple myeloma (MM) stratified for (A) race (White vs. Black), (B) gender (male vs. female), and (C) age group (66-69 years vs. 70-74 years vs. 75-79 years vs. 80-84 years vs. ≥ 85 years).

Five-year cumulative incidence of progression to symptomatic MM for SMM patients of White versus Black race was 74.1% (95% CI: 70.7%-77.1%) and 75.1% (95% CI: 65.6%-82.3%), respectively ( *P* = .75, Figure 1A). Five-year cumulative incidence of progression to symptomatic MM for male versus female SMM patients was 74.2% (95% CI: 70.0%-77.9%) and 73.3% (95% CI: 69.0%-77.1%), respectively (*P* = .32, Figure 1B). Five-year cumulative incidence of progression to symptomatic MM for SMM patients with age group 66 to 69 years, 70 to 74 years, 75 to 79 years, 80 to 84 years, and ≥ 85 years was 72.6% (95% CI: 65.4%-78.6%), 78.4% (95% CI: 72.8%-82.9%), 77.6% (95% CI: 70.7%-83.1%), 67.8% (95% CI: 60.1%-74.2%), and 68.4% (95% CI: 60.2%-75.2%), respectively (*P* = .36, Figure 1C).

Using Cox proportional hazard regression models, we found that Black, Hispanic, and other racial groups (compared to White), age ≥ 80 years at SMM diagnosis (compared to 66-69 years), an Elixhauser score ≥ 3 (compared to a score 0), the Midwest and South regions (compared to the Northeast), and state buy-in were associated with a lower risk of progression from SMM to symptomatic MM. In contrast, male gender, an age of 70 to 74 years at SMM diagnosis (compared to 66-69 years), an Elixhauser score 1 to 2 (compared to a score 0), frailty, and the West region (compared to the Northeast) were associated with a higher risk of progression to symptomatic MM although none of these differences were statistically significant (Table 2).

**Figure 2:**
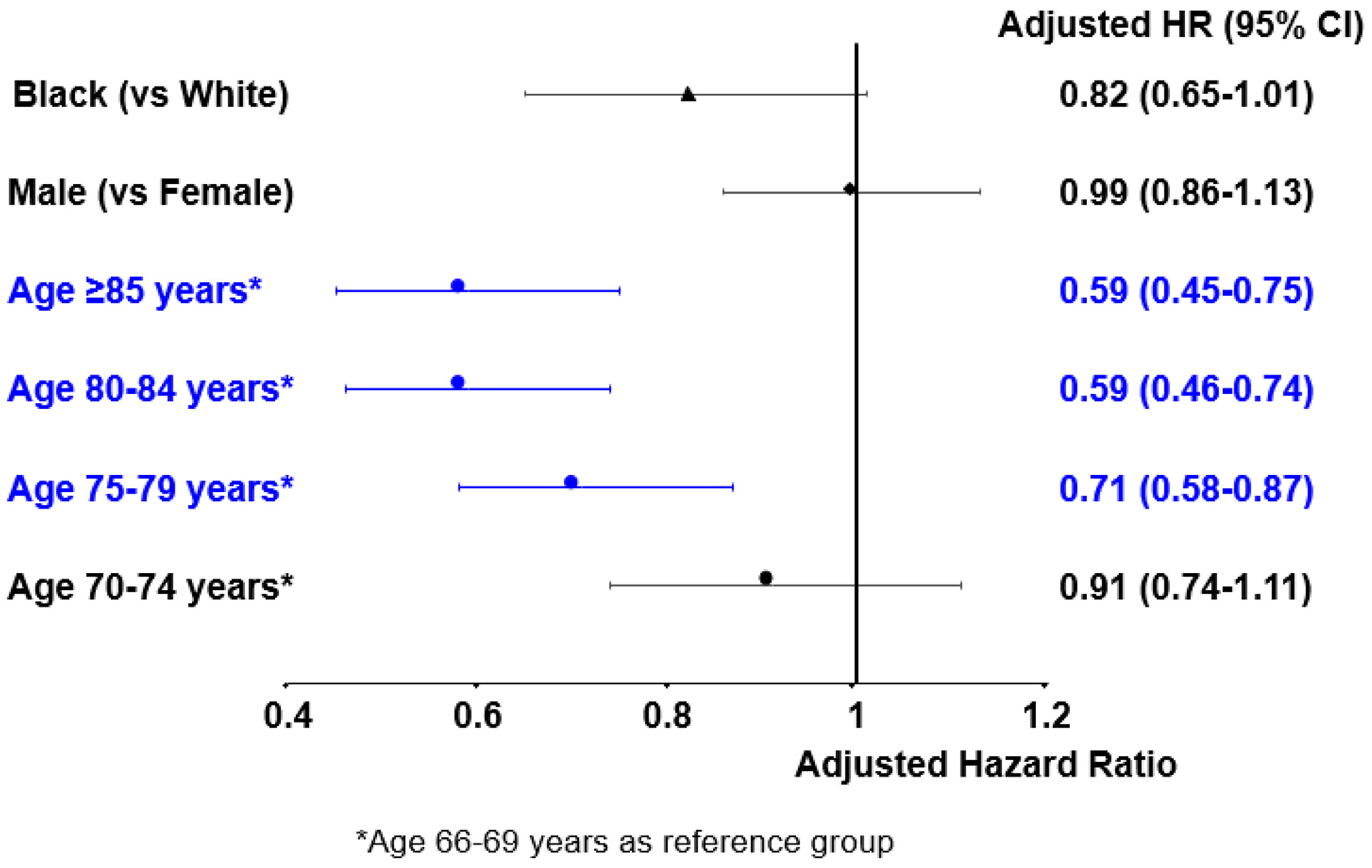
Demographics associated with progression of smoldering multiple myeloma to symptomatic multiple myeloma.

Figure 2 shows demographic factors associated with progression of SMM to symptomatic MM, adjusting for the noncancer control aHRs. Race (Black vs. White) and gender (male vs. female) were not significantly associated with SMM progression with aHR: 0.82, 95% CI: 0.65-1.01 and aHR: 0.99, 95% CI: 0.86-1.13, respectively. Only age was negatively associated with progression of SMM to symptomatic MM (age group 75-79 years vs. 66-69 years aHR: 0.71, 95% CI: 0.58-0.87; age group 80-84 years vs. 66-69 years aHR: 0.59; 95% CI: 0.46-0.74; and age group ≥ 85 years vs. 66-69 years aHR: 0.59; 95% CI: 0.45-0.75).

## Discussion

SMM is a clinically heterogeneous condition, and predicting its progression to symptomatic MM continues to pose a significant challenge. Improved understanding of the factors that drive the progression of SMM to MM will enable clinicians to provide personalized risk predictions for SMM patients, which is highly valuable in guiding decisions for early intervention and monitoring. This study provides new insights into the progression of SMM to symptomatic MM with a focus on demographic variables including race, gender, and age. Our analysis of the SEER-Medicare data, representing about 48% of the population in the United States, highlighted the unique role of age as a predictive factor for progression of SMM to symptomatic MM.

Our study found that race (Black vs. White) and gender (male vs. female) were not significantly associated with the progression from SMM to symptomatic MM. The absence of a significant gender association was further supported by the study conducted by Mateos et al.^13^ Although our data did not show a significant association between race/gender and progression of SMM, race/gender differences in progression to symptomatic MM have been suggested in a few studies.^19–21^ It is important to note that these studies had limitations, including small cohort size, reliance on a database that does not represent a true population-level dataset, or they were not exclusively focused on SMM. For example, while Dhodapkar et al.^19^ showed that African American (AA) patients with precursor conditions carry significantly lower risk of progression to symptomatic MM compared to non-AA counterparts, the study cohort comprised both MGUS and SMM patients. In another study, Covut et al.^20^ demonstrated that White race was associated with an increased risk of SMM progression to symptomatic MM. However, this cohort was derived from the NCDB, which does not accurately represent a true population-level dataset. Ravi et al.^21^ showed that male gender was a predictor of progression to symptomatic MM within 2 years of SMM diagnosis but the cohort was relatively small (*n* = 190) and limited to a single institution’s experience.

In our study, age was found to be a significant demographic factor influencing the progression of SMM to symptomatic MM. Specifically, older age groups ≥ 75 years had a lower risk of progression compared to the 66 to 69 years age group. The results from the study conducted by Covut et al.^20^ further supported this finding. Although Cowan et al.^14^ identified age as a significant predictor of progression, their study cohort included both MGUS and SMM, rather than focusing exclusively on SMM. One possible explanation for the decreased risk of progression from SMM to symptomatic MM in older patients may be driven by competing risk of high mortality due to the presence of competing health issues and comorbidities. These findings carry important implications for clinical decision-making, particularly in the context of recent shifts toward early therapeutic intervention in selected high-risk SMM patients.^30^ In elderly patients with SMM and competing comorbidities, the potential benefit of early treatment must be carefully balanced against the risks of therapy-related toxicity and overall life expectancy.

While several studies have highlighted the importance of clinical and molecular factors in predicting SMM progression,^10–14 , 21 , 31–40^ our study emphasizes the need for an understanding of the demographic factors at play. The discordance among existing risk models for predicting SMM progression further underscores the complexity of the disease and the need for more robust models that can account for various clinical, molecular, and demographic factors.^15 , 16^ By expanding these models to include demographic variables, we may be able to better stratify patients at risk for progression and develop personalized, risk-adapted approaches to treatment and monitoring.

While the strength of our study lies in the inclusion of a large cohort from a diverse geographic area using the SEER-Medicare database, several limitations must be acknowledged. First, the study population focused on Medicare beneficiaries, which may limit the generalizability of findings to younger populations or those outside the Medicare system. Second, due to the nature of retrospective data collection and potential coding inaccuracies within claims data, there may be misclassification biases. Lastly, the algorithm used in our study^24^ to identify patients with SMM has not been validated. For instance, we did not exclude patients with secondary cancers. These individuals could have received therapies typically used for symptomatic MM, potentially complicating the accurate differentiation between SMM and symptomatic MM. Future research is needed.

In summary, while demographic factors such as race and gender were not found to be significant predictors of SMM progression in this study, age was a notable factor, with older age groups showing a slower progression. This study underscores the complexity and heterogeneity inherent in SMM progression. Further research is needed to confirm these findings and explore other potential predictors, including molecular and clinical parameters. As the landscape of SMM research continues to evolve, understanding the complex interplay of demographic and biological factors will be essential for refining risk stratification models and improving patient outcomes.

### Clinical Practice Points

Only age ≥ 75 years was negatively associated with SMM progression to symptomatic MM. Race and gender were not significantly associated with SMM progression. This study highlights the importance of demographic factors in predicting SMM progression. Understanding how race, gender, and age influence the risk of progression from SMM to symptomatic MM will enable us to better stratify patients at risk for progression and develop personalized treatment and monitoring strategies.

## Figures and Tables

**Table 1:** Baseline Characteristics of Smoldering Multiple Myeloma Patients, Overall and by Progression to Symptomatic Multiple Myeloma (MM) Status

Characteristics	Overall n (%)	Progression to MM (*n* = 856)	No Progression to MM (*n* = 379)	*P*
Total	1235	856	379	
Race				
White	947 (76.7)	667 (77.9)	280 (73.9)	.26
Black	139 (11.3)	96 (11.2)	43 (11.3)	
Hispanic	77 (6.2)	47 (5.5)	30 (7.9)	
Other	72 (5.8)	46 (5.4)	26 (6.9)	
Gender				
Female	622 (50.4)	430 (50.2)	192 (50.7)	.89
Male	613 (49.6)	426 (49.8)	187 (49.3)	
Age at Diagnosis (years)				
66-69	230 (18.6)	164 (19.2)	66 (17.4)	.13
70-74	341 (27.6)	252 (29.4)	89 (23.5)	
75-79	282 (22.8)	191 (22.3)	91 (24)	
80-84	215 (17.4)	140 (16.4)	75 (19.8)	
≥ 85	167 (13.5)	109 (12.7)	58 (15.3)	
Elixhauser score				
0	413 (33.4)	301 (35.2)	112 (29.6)	.11
1-2	509 (41.2)	349 (40.8)	160 (42.2)	
≥3	313 (25.3)	206 (24.1)	107 (28.2)	
Frailty				
Yes	334 (27)	232 (27.1)	102 (26.9)	.94
No	901 (73)	624 (72.9)	277 (73.1)	
Region				
Northeast	524 (42.4)	374 (43.7)	150 (39.6)	.56
Midwest	78 (6.3)	53 (6.2)	25 (6.6)	
South	298 (24.1)	199 (23.2)	99 (26.1)	
West	335 (27.1)	230 (26.9)	105 (27.7)	
State Buy-In				
Yes	198 (16)	131 (15.3)	67 (17.7)	.29
No	1037 (84)	725 (84.7)	312 (82.3)	

**Table 2:** Hazard Ratios (HRs) of Progression to Symptomatic Multiple Myeloma Among Patients With Smoldering Multiple Myeloma (SMM) and Noncancer Patients (Control)

Characteristics	SMM Patients		Noncancer Patients^a^	
	HR (95% CI)	*P*	HR (95% CI)	*P*
Race				
White	1.00		1.00	
Black	0.97 (0.78-1.21)	0.79	1.19 (1.16-1.23)	<.01
Hispanic	0.80 (0.57-1.12)	0.19	0.96 (0.93-0.98)	<.01
Other	0.93 (0.68-1.28)	0.65	1.00 (0.97-1.03)	0.90
Gender				
Female	1.00		1.00	
Male	1.05 (0.92-1.21)	0.46	1.07 (1.05-1.08)	<.01
Age at Diagnosis (Years)				
66-69	1.00		1.00	
70-74	1.08 (0.89-1.31)	0.42	1.19 (1.16-1.22)	<.01
75-79	1.00 (0.81-1.22)	0.98	1.41 (1.38-1.44)	<.01
80-84	0.91 (0.73-1.14)	0.42	1.56 (1.52-1.60)	<.01
≥ 85	0.88 (0.68-1.14)	0.33	1.50 (1.47-1.54)	<.01
Elixhauser Score				
0	1.00		1.00	
1-2	1.04 (0.89-1.21)	0.62	1.55 (1.52-1.58)	<.01
≥ 3	0.96 (0.78-1.19)	0.70	1.90 (1.85-1.95)	<.01
Frailty				
No	1.00		1.00	
Yes	1.11 (0.92-1.34)	0.30	1.04 (1.01-1.07)	<.01
Region				
Northeast	1.00		1.00	
Midwest	0.91 (0.69-1.20)	0.51	0.96 (0.93-0.99)	<.01
South	0.97 (0.82-1.16)	0.77	0.89 (0.87-0.90)	<.01
West	1.01 (0.86-1.20)	0.90	0.95 (0.93-0.97)	<.01
State Buy-In				
No	1.00		1.00	
Yes	0.95 (0.77-1.18)	0.65	1.07 (1.05-1.09)	<.01

*Note:* All variables in the table were mutually adjusted in the Cox proportional hazard regression models.

Abbreviations: CI = confidence interval; HR = hazard ratio.

a Noncancer patients from the 5% random sample of Medicare beneficiaries.
